# The dynamic sustainability framework: addressing the paradox of sustainment amid ongoing change

**DOI:** 10.1186/1748-5908-8-117

**Published:** 2013-10-02

**Authors:** David A Chambers, Russell E Glasgow, Kurt C Stange

**Affiliations:** 1 Division of Services and Intervention Research, National Institute of Mental Health, 6001 Executive Blvd, Rockville, MD, USA; 2 Department of Family Medicine, University of Colorado School of Medicine, Denver, CO, USA; 3 Department of Family Medicine, Case Western Reserve University, Cleveland, OH, USA

**Keywords:** Sustainability, Maintenance, Adaptation, Dissemination, Implementation, Framework, Model

## Abstract

**Background:**

Despite growth in implementation research, limited scientific attention has focused on understanding and improving sustainability of health interventions. Models of sustainability have been evolving to reflect challenges in the fit between intervention and context.

**Discussion:**

We examine the development of concepts of sustainability, and respond to two frequent assumptions —'voltage drop,’ whereby interventions are expected to yield lower benefits as they move from efficacy to effectiveness to implementation and sustainability, and 'program drift,’ whereby deviation from manualized protocols is assumed to decrease benefit. We posit that these assumptions limit opportunities to improve care, and instead argue for understanding the changing context of healthcare to continuously refine and improve interventions as they are sustained. Sustainability has evolved from being considered as the endgame of a translational research process to a suggested 'adaptation phase’ that integrates and institutionalizes interventions within local organizational and cultural contexts. These recent approaches locate sustainability in the implementation phase of knowledge transfer, but still do not address intervention improvement as a central theme. We propose a Dynamic Sustainability Framework that involves: continued learning and problem solving, ongoing adaptation of interventions with a primary focus on fit between interventions and multi-level contexts, and expectations for ongoing improvement as opposed to diminishing outcomes over time.

**Summary:**

A Dynamic Sustainability Framework provides a foundation for research, policy and practice that supports development and testing of falsifiable hypotheses and continued learning to advance the implementation, transportability and impact of health services research.

## Background

As implementation science has grown [1,2], researchers have advanced from study of facilitators and barriers that influence uptake of effective programs and policies to investigations of strategies to improve uptake. However, often studies evaluate only initial intervention adoption and implementation. Sustained practice change and broader scale-up of interventions [3] rarely are investigated, often due to the constrained timeframes for research that are set by grant mechanisms, and the budgetary and political necessity of many decision-makers to take on a short-term lens.

Recently, there has been interest in understanding and influencing the sustainability of implemented interventions. While this is progress, frequently used conceptualizations of sustainability implicitly replicate assumptions and limitations inherent in the traditional research-to-practice pathway [4,5], or in its more recent conceptualization as translational research [6]. These conceptualizations of knowledge translation often assume that interventions are optimized prior to implementation, and that they are largely independent of the context in which they are delivered [7].

The presumed linear model of intervention development, efficacy testing and implementation has resulted in the development of an armamentarium of efficacious healthcare treatments, preventive strategies, and public health interventions. While these discoveries have made advances in a number of health domains, they are often difficult to implement in a myriad of practice settings and even harder to sustain over time and in many real world and low-resource settings [8]. In addition, interventions are traditionally expected to perform worse in real-world practice than in the laboratory or the rarified clinical trial setting. We argue for a new approach to sustainability that instead integrates the themes of adaptive, contextually sensitive continuous quality improvement (CQI) and a learning healthcare system with the challenge of intervention sustainment.

The purposes of this article are to explicate:

1. Evolving understandings of sustainability and of related concepts of CQI and the learning healthcare system;

2. An iterative, dynamic approach to sustainability, termed the 'Dynamic Sustainability Framework’ (DSF) that integrates and extends these concepts; and

3. Implications of this framework for research, policy, and practice.

Given the variation with which terms central to dissemination and implementation research can be used, we include a table that lays out working definitions for the central terms of this debate Table 1.

**Table 1:** Definitions of key terms used in this paper

**Term**	**Definition**
Implementation	The process of putting to use or integrating evidence-based interventions within a setting [9].
Sustainability	To what extent an evidence-based intervention can deliver its intended benefits over an extended period of time after external support from the donor agency is terminated [9].
Sustainment	The continued use of an intervention within practice [10].
Voltage drop	The phenomenon in which interventions are expected to yield lower benefits as they move from efficacy to effectiveness and into real world use (adapted from [11]).
Program drift	The phenomenon whereby deviation from manualized protocols in real-world delivery of interventions is expected to yield decreasing benefit for patients (adapted from [12]).

### Moving beyond 'voltage drop’ and 'program drift

While the traditional linear process of intervention development, derived from pharmaceutical medication development models [13], has often resulted in the creation of initially successful interventions, it may be less helpful in enabling these innovations to maximally benefit health. A linear approach may be particularly challenging to apply to complex, multi-component interventions, psychosocial treatments, treatment of the growing number of people with multimorbid conditions [14,15], and systemic approaches to care [15]. Linear approaches place a premium on creating and 'freezing’ an intervention, developing manuals to ensure its consistent delivery with fidelity [16], and then minimizing deviations from the intervention. For example, the COMMIT stop smoking national project had expert researchers design the complex, lengthy intervention protocol based on research evidence, and then gave a several hundred page manual of operations to local staff to implement in their communities [17].

**Figure 1:**
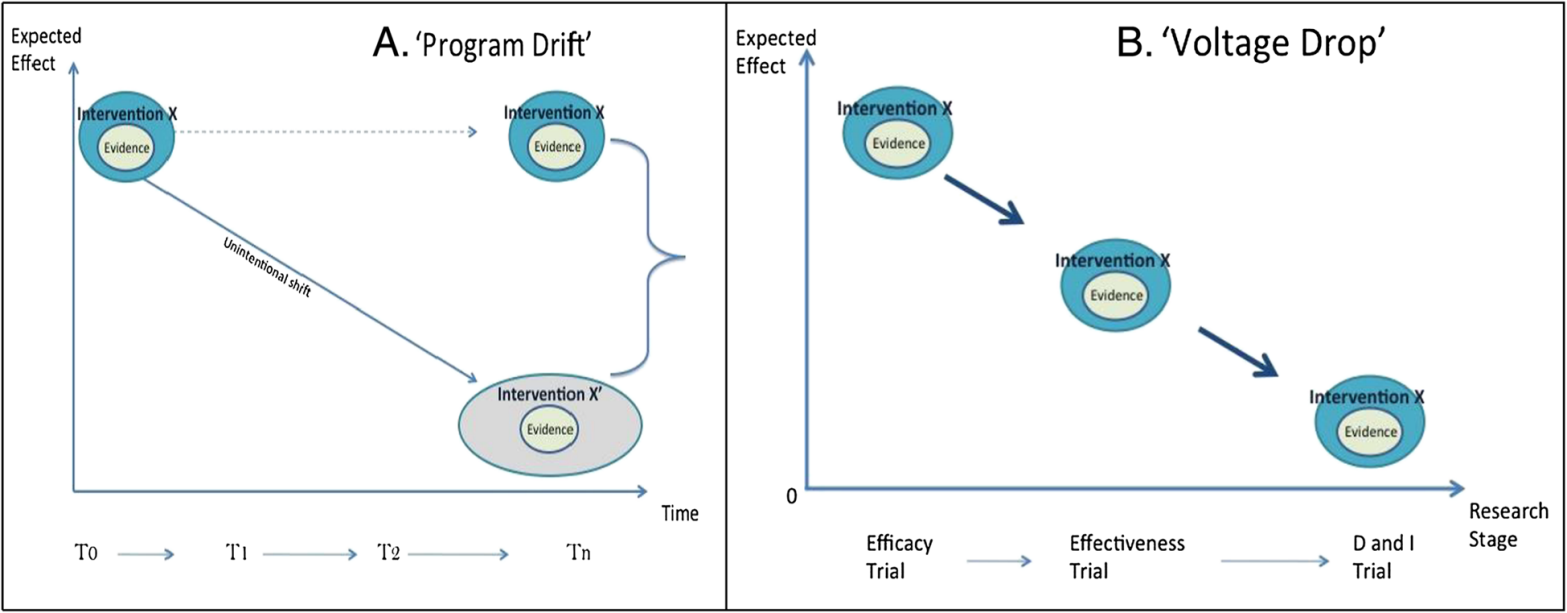
**Program drift and voltage drop.** Illustrating the concepts of 'program drift,’ in which the expected effect of an intervention is presumed to decrease over time as practitioners adapt the delivery of the intervention **(A)**, and 'voltage drop,’ in which the effect of an intervention is presumed to decrease as testing moves from Efficacy to Effectiveness to Dissemination and Implementation (D&I) research stages **(B)**.

The importance of internal validity to the scientific process should not be ignored, but its overemphasis relative to generalizability and adaptation runs the risk of creating interventions that will not fit within different, complex or changing settings and of failing to benefit settings, clinicians, and patient populations who are underrepresented in the intervention testing process [7,16]. Two key implicit assumptions within the traditional intervention development approach may limit ultimate progress toward intervention sustainability and population impact:

First, interventions are often developed with the idea that they can be optimally constructed, manualized, and then tested in a single form applicable across settings and over time. Efficacy trials are designed to screen out noise in the system (patient comorbidities, competing demands and skill variance of clinicians, resource limitations, varying motivations of patients) [7], and thus maximize outcomes. As interventions move to effectiveness and into implementation, one expects that the individual benefit of the intervention will likely drop, due to the added complexity of heterogeneous patients, providers and settings. This is referred to as 'voltage drop’ (Figure 1). The assumption of 'voltage drop’ results in missed opportunities to refine and improve the intervention, instead concluding that the declining benefit is expected and acceptable, and the best possible outcome is that which is achieved at the efficacy stage.

Second, the assumption that interventions can be optimally constructed in the early stages of the development and testing process, independent of context, suggests that, even at the stages of implementation and sustainability, change to the intervention is expected to have negative consequences, and that the further a practitioner deviates from the manual, the lower the benefit. This is the concept of 'program drift’ (Figure 1). Delivering the intervention within an efficacy trial may require adherence to protocols that are challenging to deliver within real-world practice. Fidelity ratings then assume that 100% fidelity to original protocols will yield optimal outcomes, and effort is expended to ensure that practitioners do not deviate from the manual. Where clinicians do deviate from the protocol, the field expects that the resulting 'program drift’ will compromise outcomes [12]. We see that this over-reliance on quality assurance to prevent 'program drift’ leads to extensive pressure on real-world practices to adhere to the intervention protocols without evidence that this adherence will lead to optimal outcomes. Quality assurance may inadvertently hamper sustainability and ongoing improvement, customization and optimization of interventions to the detriment of population health.

In contrast, we reject the notion that an intervention can be optimized prior to implementation, and explicitly reject the validity of 'program drift’ and 'voltage drop.’ Rather, we suggest that the most compelling evidence on the maximal benefit of any intervention can only be realized through ongoing development, evaluation and refinement in diverse populations and systems [18]. Instead of viewing contextual factors as interfering with the delivery of an effective intervention and needing to be controlled, we see the opportunity to learn about the optimal fit of an intervention to different care settings [2]. For example, strategies have been developed to adjust organizational characteristics (*e.g*., culture, climate, structure) to enable improved fit between the intervention and the setting [19]; harnessing the understanding of context can enable beneficial adaptation of the intervention and improve sustainability. Without rejecting these assumptions, we reify early phase interventions tested in the most artificial settings, set quality assurance of interventions as an optimal outcome, and miss opportunities for continued learning and development.

### Understanding and advancing sustainability research

As the field of implementation science has matured [20,21], more emphasis has been placed on understanding sustainability. Researchers have recognized that implementation of interventions, which can often require substantial resources, is meaningless without successful long-term use. Following Rabin *et al.*’s glossary for Dissemination and Implementation Research in Health [9], we draw distinctions between 'implementation,’ which relates to the initial process of embedding interventions within settings and 'sustainability,’ which relates to the extent that these interventions can continue to be delivered over time, institutionalized within settings, and have necessary capacity built to support their delivery.

Recent articles [22-24] have advanced the idea of an adaptation phase that bridges from the initial implementation effort to a longer-term sustainability phase. They argue the need to examine the fit between the practice setting and the intervention and make changes necessary to improve the integration of the intervention into ongoing care processes. This is consistent with the institutional theory of organizations, which argues that the final stage of innovation requires the 'institutionalizing’ of the new practice so that it becomes a working part of the organization [25].

As a consequence, assessment of organizational characteristics (*e.g*., structure, climate, culture, resources) is seen as an essential component of sustainability, and indeed, the fit between context and the intervention is at the center of a sustainability phase [24]. There has also been an emphasis on planning for sustainability much earlier in the intervention process [26]. Recent approaches to sustainability locate key efforts squarely in the implementation phase, arguing that once a practice has been implemented within a care system, those who manage the delivery of that practice should turn their attention to ensuring that the practice can be maintained over time [6,24]. Authors typically suggest that this entails attention to issues of long-term financing, training of the workforce, supervision, and organizational support for the practice [26,27]. A characteristic of this approach is to postpone emphasis on sustainability until after implementation is well underway, assuming that implementation and sustainability are sufficiently independent.

Authors have also highlighted the utility of assessing outcomes of those who have received the practice, something infrequently collected in routine practice [24,28]. Seldom is it demonstrated that continued delivery of an intervention confers benefit on the patient population that receives the intervention or the system that delivers it (*e.g*., cost containment, efficiency of care, quality metrics). Measurement of outcomes over time to determine continued benefit has been shown to support sustainability of the practice [29,30].

Recent implementation projects have created new tools and scales to study sustainability, including needs assessments, long-term action plans, tracking of program adaptation, financial planning, mapping of community networks, and measurement of the degree to which practices are integrated and institutionalized into service systems [31-33]. While this emerging focus on sustainability is an advance, many studies still assume a largely static service delivery system that needs to be assessed only at key time points, but not in an ongoing manner. To better reflect complexity and change within the system and in context, a more dynamic approach to sustainability is needed.

### Framework

#### The dynamic sustainability framework (DSF)

**Figure 2:**
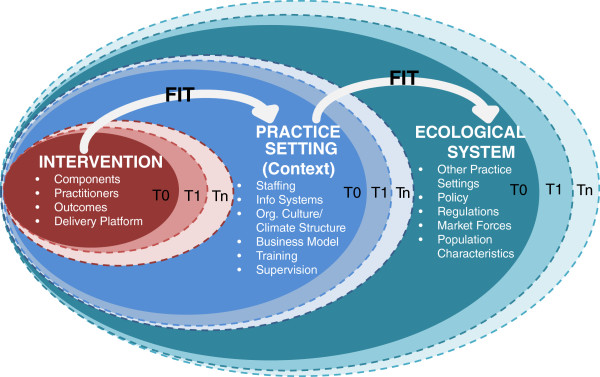
**The dynamic sustainability framework.** Illustrating the goal of maximizing the fit between interventions, practice settings, and the broader ecological system over time (represented by T_0,_ T_1,…,_T_n_), each of which has constituent components that may vary.

As Heraclitus observed, 'The only constant is change.’ The Dynamic Sustainability Framework has developed out of our evolving thinking and our collective experience in conducting and advancing implementation science, where we have seen inattention to constant change limit the ability to which implemented interventions are sustained over time in complex clinical and community settings. The DSF (Figure 2) emphasizes that change exists in the use of interventions over time, the characteristics of practice settings, and the broader system that establishes the context for how care is delivered. As classical thinking eloquently captures, change impacts the ability of health interventions to be optimally used and sustained over time. This dynamism exists in the evidence base for interventions that links causal factors to health outcomes, as judged by the continual stream of new publications in academic journals that add to available evidence on the effectiveness of interventions, as well as ongoing practice surveillance systems that capture intervention impact. Dynamism exists in the interventions that support the evidence, which acknowledge ad hoc adaptation and experimentation of evidence-based interventions. Furthermore, it exists in a constantly changing multi-level context [34], internal to a clinical or community setting and the broader care system, be it an organization, community, county, state or country.

The DSF, like many implementation models, centers on a few major elements: the intervention, the context in which the intervention is delivered, and the broader ecological system within which the practice settings exist and operate. Distinct from those models, however, is the consideration of these elements over time. The intervention, as shown in the figure, often includes a set of individual components chosen for their ability to effect behavior or biochemical change, an assumed set of characteristics defining who should deliver the intervention, targeted, patient-centered outcomes that the intervention should generate as a result of its use, and a delivery platform (*e.g*., face-to-face, telephonic, web-based, mobile health app, etc.). Other constructs may also define the intervention.

The DSF anchors the ultimate benefit of the intervention in terms of its ability to fit within a practice setting, typically a clinical or community setting. This context carries its own set of characteristics, including human and capital resources, information systems, organizational culture, climate and structure, and processes for training and supervision of staff. The DSF, consistent with other models, argues that these practice characteristics will directly influence the ability of the intervention to reach the patient population that could benefit, and thus measurement of these contextual constructs is paramount to resolving fit.

At a third level, the DSF identifies the ecological system as an additional driver of the successful implementation and sustainability of an intervention. The ecological system consists of many practice settings that influence those working to incorporate a particular intervention, as well as the legislative and regulatory environment, characteristics of local, regional, state and national markets, and characteristics of the broad population. The ecological system is influenced by changes to available interventions and practice settings, and in turn, influences them.

Specific to the DSF, as emphasized by the dotted lines in Figure 2, is the expectation that change is constant at each of these levels (and ripples across multiple levels), and thus the success of an intervention to be sustained over time lies in the measured, negotiated, and reciprocal fit of an intervention within a practice setting and the practice setting within the larger ecological system. The DSF suggests that optimal fit requires that characteristics of the intervention, practice setting, and ecological system be consistently tracked, using valid, reliable and relevant measures, and expects that interventions, settings and the ecological system should change over time, particularly where data can suggest improvements for each to better meet the needs of patients, the skills and resources within the practice setting, and the larger ecology.

The DSF is intended to suggest a new paradigm to consider the long-term use and ongoing improvement of interventions, recognizing the limitations of the evidence base made available through efficacy and effectiveness trials, and allowing that continuous exposure of the intervention to new populations, new contexts, and new innovations can result in continued improvement of resulting outcomes, thus minimizing the perils of 'program drift’ and 'voltage drop.’ Indeed, the DSF posits that ongoing quality improvement of interventions is the ultimate aim, not quality assurance of them. To be clear, we see value in ensuring appropriate quality assurance within healthcare systems where clear assessments of an appropriate standard of care are made through knowledge of core intervention components. However, the DSF recognizes the limitations of intervention evidence solely from clinical trials and argues that quality improvement processes focused on intervention optimization are ultimately more relevant to achieve sustainment.

The DSF, which has benefitted from the authors’ ongoing dialogue with the Implementation Science community about the challenge of sustainability, follows the spirit of a number of existing models that emphasize three things—importance of context, the need for ongoing evaluation and decision-making, and the goal of continuous improvement. These include Wandersman’s Getting to Outcomes model [31], Continuous Quality Improvement (CQI) [34], system dynamics [35], complexity theory [36], adaptive management [37], and the Evidence Integration Triangle [30]. In addition, the DSF is consistent with alternative views of organizational development [38] and the principles of system science [39]. Distinct in the DSF from many of these other models is the emphasis on omnipresent change, and the central goal of continuously optimizing the fit between the intervention and a dynamic delivery context to achieve maximal benefit. The DSF is anchored around the following seven tenets, for which we think there is evidence, but recommend explicit testing in this context:

### An intervention should not be optimized prior to implementation, or even prior to 'sustainability phase’ onset

Interventions benefit from ongoing optimization as they are applied in different contexts [37]. The evidence that supports the benefits of health interventions arises from trials that represent a very small slice of the diversity of demographics, preferences, and health status of the population at large [7,40], but we should not expect evidence collected in one set of narrow, relatively optimal circumstances to apply perfectly in other, vastly different contexts [41]. A 'corollary’ of this recommendation is that, other things being equal, quality improvement approaches that involve adjusting and refining program should be more effective than 'quality assurance’ procedures that emphasize fidelity to an initial protocol.

### Interventions can be continually improved, boosting sustainment in practice, and can enable ongoing learning among developers, interventionists, researchers and patients

There is tremendous opportunity to aggregate evidence on the real-world impact of interventions when used in practice. We can apply models of continual refinement that have been the cornerstone of software development (*e.g*., Firefox, Reaper, iTunes), as well as Web 2.0 sites (*e.g*., Wikipedia, Facebook, etc.) [42]. By augmenting trial data with practice-based evidence, we can understand much more about what works for whom, the question underlying personalized medicine [43,44]. This articulation of the DSF suggests the need for a long-term plan to commit resources for training and ongoing improvement. One implication of the DSF is that intervention impact can also be enhanced through increases in efficiency. The field has developed a plethora of multi-component interventions, often without studies that determine what the minimal set of components are needed to ensure benefit [45]. The DSF, congruent with the Consolidated Framework for Implementation Research [46], emphasizes the importance of streamlining interventions to peel away components that may not be central to improving outcomes or to adapt intervention components to a particular context.

### Ongoing feedback on interventions should use practical, relevant measures of progress and relevance

Too often, intervention trials focus on markers that are psychometrically valid but of less relevance to patients and clinicians [3,47]. For example, very specific, intervention-related symptomatic scales may be most sensitive to change, but there is little guarantee that the measured change translates into a tangible, functional benefit for the patient [48]. The DSF thus suggests the use of measures such as checklists that are relevant to desired outcomes of patients, as well as sensitive to the 'fit’ between interventions and context, and can be feasibly implemented [49]. Across each of the changes in Figure 2, we see available streams of data that can offer leverage points for improving interventions. Environmental changes, for example, can be tracked via population surveys, and market and claims data. Practice changes can be captured through electronic health records, claims data and practice surveys. Evidence reviews can provide key information on knowledge changes, and policy changes can be tracked via available Federal and Non-Profit sources (*e.g*., CMS, Kaiser Family Foundation, etc.). This is consistent with the CQI model, but with specific emphasis on the ongoing refinement of the intervention to counteract the assumptions of 'Program Drift.’

### Voltage drop is NOT inevitable

We reject the assumption that the more diverse and complex a patient population is, the smaller the benefit of intervention, referred to above as 'voltage drop’ [11]. This stems from an expectation that intervention studies require control of the environment to isolate a treatment effect [7]. If we embrace CQI of the specific health intervention, we expect that with more experience, we will better be able to adapt interventions to contexts and patients. As we learn more about what works and what doesn’t and adjust protocols accordingly, the 'voltage’ could maintain or possibly increase over time. This echoes the computer industry, where each new release of a hardware or software line is expected to be better than the prior version. It also finds consonance with the evolution of the flu vaccine, which is constantly refined in response to the changing nature of the influenza virus each season. A culture of improvement is central to ongoing intervention use and treats improvement of the intervention as central to the sustainability process [33].

### Programs should be more likely to be maintained when there is strong 'fit’ between the program and the implementation setting

The concept of 'fit’ has been discussed by other authors (*e.g*., Estabrooks, Glasgow, Dzewaltowski), [50] and goes back at least as far as Rogers’ *Diffusion of Innovations*[51], where the concept of reinvention evoked the notion of departing from the original intervention concept to 'create’ a new version suited to the preferences and constraints of the local context. Fit is a multi-level construct and involves alignment along multiple dimensions [50]. The DSF posits that fit will likely change over time, due to changes in the way in which an intervention is delivered, the characteristics of patients, providers and settings, and the broader ecological system within which healthcare settings reside. Attention to this fit, through ongoing assessment and quality improvement efforts, should improve sustainment and ultimately identify opportunities for intervention improvement.

### Organizational learning should be a core value of the implementation setting

While training and ongoing analyses exist in many organizations, the demands imposed by multiple levels of change require learning to be central to organizational activity for interventions to become sustainable. The context both within an organization and in the broader ecological system is constantly changing, and requires a 'learning organization’ [52] to engage in problem-solving capacity at multiple levels [53]. Organizational learning should also target appropriate adaptation of evidence-based interventions, possibly in rapid learning cycles [44,54], followed by ongoing assessment and feedback loops. The DSF is congruent with the concepts of the learning healthcare system, again with the emphasis on learning how to better develop, deliver and sustain interventions.

### Ongoing stakeholder involvement throughout should lead to better sustainability

Continuously engaging stakeholders throughout the planning, implementation and adaption processes should help increase the fit between the intervention and the local context, and help address evolving issues that might interfere with sustainability. Just as researchers have proposed intervention development processes [12] that focus on the ultimate site where interventions will be delivered, we argue that partnership among all relevant stakeholders is essential to maintaining and improving interventions within care settings.

**Figure 3:**
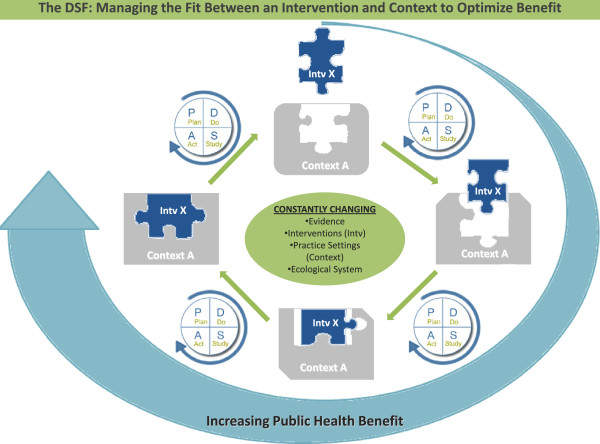
**Using the dynamic sustainability framework as an engine for quality improvement.** The DSF depicts a dynamic view of sustainability, which allows for the evolution of an intervention within a changing delivery system. The changes in the shape of the puzzle pieces and of the contexts reflects the ongoing change to interventions, practice settings, and care systems, and shows the use of quality improvement methods to optimize the 'fit’ and improve the public health benefit of sustained use of interventions.

As Figure 3 depicts, we view sustainability as akin to the challenge of fitting a puzzle piece within an evolving large tableau. Without sensitivity to the characteristics of the intervention, practice setting and the larger system, there is little expectation that the intervention will fit well within the setting, and as the context changes (noted by the changing shape in the figure), sustainment will be harder and harder to achieve. However, by examining and adapting the intervention to a changing context, we believe that sustainment is not only possible, but that the utility of the intervention can be optimized. The experience of delivering the intervention in vivo over time serves to inform the ongoing evolution of the intervention (noted by the change in shape of the puzzle piece). By concentrating on the dynamic 'fit’ between an intervention and its delivery context as the core ingredient underlying sustainability, we embrace opportunities to refine and improve the intervention.

### Contrasting static and dynamic views of sustainability

Table 2 compares static views of sustainability with the DSF, offering sharp contrasts in data collection and analysis, opportunities for knowledge development and incorporation of the 'noise’ within healthcare contexts. Rather than seeking to simplify the phenomenon of study, either by avoiding adaptation of interventions, or assuming the context to be unchanging, the DSF embraces change as a central influence on sustainability. Adaptation is expected, and even encouraged. Assessment of care settings and outcomes is ongoing and incorporated within practice, and staffing and policy changes are incorporated in sustainability planning. Perhaps the biggest contrast of the static and dynamic views is that the static view limits lessons that can in turn provide feedback to other areas of science; the DSF views an abundance of ongoing evidence that can be cycled to continuously improve intervention design, testing, and ongoing system change.

**Table 2:** Contrasting static views of sustainability with the dynamic sustainability framework

	**Static view**	**Dynamic sustainability view**
Adaptation	Bad; avoided/eliminated	Inevitable; encouraged, monitored and guided by evidence
Context assessment	Initial or during implementation	Ongoing
Outcomes assessment	During study by researchers	Incorporated as part of organization
Review of evidence	Initial- from efficacy studies	Ongoing; from convergent sources including replications
Staffing issues (*e.g*., turnover) and variations	Ignored/feared	Planned for; investigated
Generates new knowledge	No	Yes, feedback to other areas of science and to earlier stages

*Note.* This table contrasts more traditional static views of sustainability, in which efforts are made to minimize change and retain the original form of an intervention, from a dynamic sustainability view that we suggest in the DSF, in which change is inevitable and can lead to better fit and ultimately better impact of interventions.

## Discussion

### Implications of the DSF

This initial formulation of the DSF has implications for future practice, research and policy. For practice, the DSF highlights the need for continuous assessment of the local context, not just prior to implementation. This enables care settings to better manage the fit between their resources, needs and the interventions, including generating consistent feedback on how interventions are delivered to diverse patients and how patients do as a result. The collection and analysis of this information allows practitioners to make informed decisions about how best to utilize existing interventions, allows for potential enhancements to the interventions to be made and shared, and offers better information on which to make decisions to cease delivering interventions that do not have benefit. The intention is to recognize and support rapid learning, real-time problem-solving organizations [54] that are full partners in the generation of knowledge, not just its application. Thus, the DSF promotes the use of multiple methods of planning for sustainability, including simulation modeling of the impact of different decisions, pilot testing of adaptations within local contexts, and continued experimentation. Perhaps an even greater benefit to practice would come through pooling of data across a larger set of sites, practitioners and patients, something done with success for chronic disease [55,56].

For research, the DSF dispels the notion that intervention development, refinement and improvement are completed prior to real world implementation. In contrast, we suggest that development and refinement is never complete. Rather, sustainability is the process of managing and supporting the evolution of an intervention within a changing context. We recommend (and welcome testing of the idea) that programs that monitor context and adjust accordingly do better long-term. In addition, we see research studies testing whether settings and programs using ongoing CQI or other means of feedback improvement perform better over time. More broadly, we see the DSF as changing the notion of a linear transition from research to practice into a shared process of continual experimentation and analysis through the use of both practice settings and ecological systems to track changes and assess evolving fit between interventions and practice settings. Principles of 'crowd sourcing’ made popular within the IT industry and resulting in open source products like Firefox, Wikipedia and Reaper, blur the lines between research and practice. We see this in the evolution of the electronic health record, and the rapidly evolving patient health record, which is dramatically changing the scenario from one where the medical expert makes all the decisions to one of collaborative care and shared decision-making [57,58]. Instead of a small team of researchers developing a priori an 'optimal,’ static product, a large and often virtual community including users and consumers continuously upgrades dynamic products.

This initial formulation of the DSF also has implications for policy. Incentives are needed to support ongoing adaptation of interventions, particularly where evidence is limited, specifically including monitoring of progress and documentation of adaptations, using quality measures relevant to stakeholders and patients. In addition, research funders must determine how to support longer-term projects related to sustainability with flexible research designs, since the ultimate benefit of integrating and modifying interventions may not be evident for many years. In addition, infrastructure to support pooling of 'practice-based evidence’ will be needed, in order to ensure sufficient information is available about long-term use and adaptation of interventions. The DSF aligns directly with a number of existing policy initiatives, at national, state and local levels, including the advance of Patient-Centered Medical Homes, Accountable Care Organizations, Pay for Performance initiatives, and support for local demonstration projects.

We recognize that this conceptualization of the DSF model should, consistent with its internal logic, be refined and improved over time. Whether this happens through testing of the tenets laid out in the previous section or contributions of others’ theoretical or empirical studies, we offer the DSF as the beginning of a longer debate. For example, while Figure 2 shows three levels (intervention, practice setting, ecological system), we appreciate that many more levels of the system exist than what we have depicted in the figure. We see further specification of the interrelationships of those levels as a useful area for further research and development. In addition, we see the utility of aligning the DSF with alternative methods of developing and testing dissemination and implementation interventions from the prevailing linear model. In an effort to present examples of how the DSF might be used to consider the sustainability of various types of interventions, we have included Table 3.

**Table 3:** Illustrative examples of the use of DSF for different types of interventions

**Intervention example**	**Applying DSF principles**
**Clinical guidelines for pharmacotherapy** • Clinical guidelines for pharmacotherapy to treat a range of chronic diseases have been developed, implemented and refined as new compounds have reached the market and new evidence has been gathered about the relative benefit of different medications. • The influences on prescribing practices exist at the patient level (preferences and predictors of response), clinician level (practice patterns, level of training), system level (formulary design, insurance coverage, adherence monitoring). • Each influence will impact guideline implementation and overall benefit of care for patients served within the health system.	• Assessing appropriate fit between guidelines and the care setting will require analysis of multiple streams of data, including administrative, clinical, organizational and epidemiologic. • The DSF suggests that collecting benchmarks over time on patient outcomes, adaptations in algorithms used, available evidence (from the literature, healthcare systems and patient populations), and contextual factors could result in improvements to the guidelines, to the capacity of the health system to more seamlessly integrate the guidelines, to the ecological system that could improve access, quality and health outcomes.
**Psychotherapy for mood disorders** • Manualized evidence-based psychotherapies for mood disorders have been tested in numerous studies. • Many of these therapies are designed to be delivered by specific providers, over a set number of sessions, with a clear step-by-step approach. • Given variation in access to therapy (*e.g*., number of sessions covered, availability of therapists, time), limited predictive ability of response (how many sessions are needed, what are active ingredients, who should deliver therapy?), emergent options for mode of delivery (web-based, face-to-face, self-guided, asynchronous), optimizing psychotherapy for individuals and systems is still beyond our current knowledge base.	• The DSF suggests manualized psychotherapy could be improved by tracking variation in use and therapeutic response of patients, contextual characteristics that influence delivery, and additional interventions that affect clinical and functional outcomes. • Systems could track how patients respond to varying doses of therapy, modes of delivery, and clinician characteristics. Over time, decision-makers could align available care to the needs of patient populations, and clinicians could adapt practice patterns to data on patient preferences and outcomes, and general needs of the patient population. • By assessing the fit of psychotherapy delivery with patients, the service setting and the broader ecological system, the DSF hypothesizes that new insights about psychotherapy optimization could drive improvements in patient care.
**Care management for chronic diseases** • Studies have shown the effectiveness of care management strategies to assess, intervene and monitor for a range of chronic conditions. • Typical strategies involve initial screening, assessment, treatment planning, care and self-management strategies, and follow-up. • While general care management approaches seem to be durable, specific approaches can have difficulty being implemented across many clinical and community settings, because of limitations in resources (both monetary and staffing), information systems, financing processes, and other barriers. • With new technologies, additional evidence about treatment and preventive interventions, and reconfiguration of care systems, care management for chronic diseases can be sustained and improved in a large variety of care settings.	• Care management is influenced by drivers at patient, provider, organization and system levels. • Care management requires coordination among multiple people, organizational supports and capital resources, all of which will likely shift over time. • Therefore, care management cannot be sustained without continual assessment of fit within the local setting and the support of the components of the model. • The DSF hypothesizes that attention to local adaptations made by healthcare and community settings to fit the model; and feedback on staffing levels, intensity of care management, emerging interventions and patient outcomes could enhance long-term sustainability and model improvement. • Evidence about who benefits most from different variants of care management, who is an ideal care manager, and what are the best ways of coordinating across primary care and specialty practice could lead to better uptake and improve patient health.

*Note.* This table offers examples of how the sustainability of different types of interventions can be enhanced by applying the principles of the DSF. Both the descriptions of the interventions and the use of the DSF to improve them are exemplars to further the debate, and are not intended to comprehensively depict the extensive levels of influence.

## Summary

It is time to embrace the culture of a learning healthcare system [44,59] to promote sustainability of interventions that are optimized and customized to the myriad of clinical and community settings. The enormous changes in health systems in the past few years give particular salience to a conceptualization of sustainability as a dynamic process, and provide unparalleled opportunity to test and refine the principles offered in this paper. Without this emphasis, we reify the past by asserting the primacy of evidence-based guidelines based on evidence from rarified clinical trial settings, and give second-class status to ongoing learning in real world settings. Allowing researchers to be the only ones allowed to generate new knowledge limits the opportunities to consistently improve the care we provide. In the past, these limitations were technological; we lacked the tools and the sources of information to drive rapid improvements. In this era of 'crowd sourcing,’ of exponentially-expanding processing power and global connectedness, we no longer need to adhere to a view that once created, interventions and healthcare settings must be 'frozen’ to optimize effectiveness. Instead, we propose the DSF as helping to reconfigure the research-practice-policy interface, in which the best possible information is gathered and used in real-time to inform policy, improve practice, and answer the highest priority research questions. Only then will the promise of a learning healthcare system be reached.

## Competing interests

REG is a member of the Senior Advisory Board of *Implementation Science*; all decisions regarding this paper were made by the editors. The authors declare no other competing interests.

## Authors’ contributions

DAC conceptualized and wrote the manuscript. REG and KCS provided input on the conceptual framework and edited the manuscript. All authors read and approved the final manuscript.
